# Anti-Fatigue Effects of Donkey Milk and Its Polysaccharides in a Mouse Swimming Model

**DOI:** 10.3390/foods15142568

**Published:** 2026-07-22

**Authors:** Teng Wang, Yangen Sun, Kaiwen Wang, Chuanliang Ji, Gengli Huang, Jie Xiao, Mengyan Zhang, Jingwen Zhou, Xiaoshu Tang, Xuemei Chen, Bo Hu, Jie Yu, Zhouping Wang

**Affiliations:** 1 State Key Laboratory of Food Science and Resources, Jiangnan University, No. 1800 Lihu Avenue, Wuxi 214122, China; 2 School of Food Science and Technology, Jiangnan University, No. 1800 Lihu Avenue, Wuxi 214122, China; 3 National Engineering Research Center for Functional Food, Jiangnan University, No. 1800 Lihu Avenue, Wuxi 214122, China; 4 Shandong Key Laboratory of Gelatine Medicines Research and Development, Dong’e Ejiao Co. Ltd., Liaocheng 252200, China; 5 National Engineering Technology Research Center for Gelatin-Based Traditional Chinese Medicine, Liaocheng 252200, China

**Keywords:** donkey milk, polysaccharides, monosaccharide composition, antioxidant activity, anti-fatigue

## Abstract

Exercise-induced fatigue is a physiological limitation affecting athletic performance and recovery, driven by disrupted energy metabolism, oxidative stress, and inflammation. This study investigated the anti-fatigue effects of donkey milk and its polysaccharides in BALB/c mice using an exhaustive swimming model. Mice were randomly assigned to seven groups, including a negative control group and separate dose groups receiving donkey milk (2.08, 4.16, or 8.32 mL/kg) or donkey milk polysaccharides (0.625, 1.25, or 2.5 g/kg), for 30 days. Anti-fatigue activity was subsequently evaluated based on exhaustive swimming performance and fatigue-related biochemical parameters. Donkey milk polysaccharides, mainly composed of galactose, exhibited stronger in vitro antioxidant activity than donkey milk, which may be associated with their enhanced radical scavenging capacity. Both treatments significantly prolonged exhaustive swimming time (up to 2.27-fold, *p* < 0.05) without affecting body weight. They improved energy metabolic status (glycogen, ATP, lipid utilization), reduced fatigue-related metabolite accumulation, enhanced antioxidant capacity, and suppressed oxidative stress and inflammatory responses, with donkey milk polysaccharides generally showing superior efficacy. The in vitro antioxidant activity was consistent with in vivo improvements in oxidative status, supporting a functional link between polysaccharide bioactivity and physiological effects. These findings suggest that donkey milk and its polysaccharides may alleviate exercise-induced fatigue through coordinated regulation of energy metabolism, oxidative stress, and inflammation, providing a mechanistic framework linking polysaccharide composition to anti-fatigue effects.

## 1. Introduction

Exercise-induced fatigue is a multifactorial physiological condition resulting from the complex interplay among energy depletion, oxidative stress, and inflammatory activation, ultimately compromising physical performance and recovery when persistent [1,2]. High-intensity or excessive exercise markedly increases the production of reactive oxygen species, disrupting redox homeostasis [3]. Depletion of hepatic glycogen (HG) diminishes muscle contractile efficiency and limits endurance capacity [4], while reduced adenosine triphosphate (ATP) synthesis accelerates accumulation of metabolic byproducts, including blood lactate (LA) and blood urea nitrogen (BUN). The resulting acidosis disrupts muscle function and ionic homeostasis, further exacerbating fatigue [5,6]. Additionally, an imbalance between mitochondrial biogenesis and degradation during high-intensity exercise impairs reactive oxygen species regulation, accelerates the decline in contractile efficiency, and contributes to metabolic fatigue [7,8,9,10,11]. Excessive exercise also elevates pro-inflammatory cytokines and activates immune cells, which exacerbate muscle damage, delay recovery, and intensify fatigue [12]. Therefore, mitigating exercise-induced fatigue is a major research priority, and identifying safe nutritional interventions represents a promising strategy. Nutritional strategies capable of simultaneously regulating oxidative stress, energy metabolism, and inflammatory responses have therefore attracted increasing attention [1,2].

Specialty dairy products have long been consumed as traditional dietary staples, and their safety and richness in bioactive compounds have recently positioned them as promising anti-fatigue functional foods [13]. Donkey milk, a traditional nutraceutical, possesses a protein and polysaccharide composition similar to human milk, with whey protein accounting for approximately 40% of total protein, along with high lysozyme content and other bioactives [14,15,16,17]. Its distinctive nutritional and bioactive attributes have attracted attention for diverse applications, including allergy alleviation, immunomodulation, dermatological improvement, antioxidant, anti-inflammatory, anti-aging, anti-tumor, and anti-fatigue activities, as well as metabolic disorder interventions [18,19,20,21,22,23,24,25,26,27,28,29,30]. The lactose content of donkey milk (58–74 g/L) closely resembles that of human milk (63–70 g/L) and is higher than that of other milk sources [17]. Moreover, donkey milk oligosaccharides promote intestinal epithelial cell maturation, maintain epithelial stability, and support intestinal development [31]. Accumulating evidence suggests that donkey milk and its polysaccharides can stabilize blood glucose, replenish energy substrates, increase glycogen reserves, and scavenge free radicals, thereby alleviating exercise-induced fatigue [13,32]. Specific sialylated oligosaccharides in donkey milk further enhance calcium and phosphorus absorption, improve trabecular microarchitecture, and exert prebiotic and broad-spectrum antimicrobial effects [16]. These pleiotropic actions indirectly sustain energy metabolism and consequently mitigate fatigue. Nevertheless, current studies have mainly focused on isolated bioactivities of donkey milk and donkey milk polysaccharides, such as antioxidant, anti-inflammatory, and myofibrillar protective effects. However, their potential anti-fatigue effects and the associated physiological mechanisms linking energy metabolism, oxidative stress, and inflammatory responses remain insufficiently elucidated. This study was designed to address this gap.

This study systematically evaluated the antioxidant and anti-fatigue activities of donkey milk and its polysaccharides. We characterized the molecular-weight distribution and monosaccharide composition of the isolated polysaccharide fraction, and assessed their antioxidant potential in vitro and anti-fatigue effects in vivo using a mouse exhaustive swimming model. These findings provide an integrated experimental framework linking polysaccharide structure, in vitro antioxidant activity, and in vivo physiological effects. This may provide experimental evidence supporting the potential role of donkey milk polysaccharides in modulating energy metabolism, oxidative status, and inflammatory responses during exercise-induced fatigue.

## 2. Materials and Methods

### 2.1. Materials and Reagents

Fresh donkey milk was provided by Shandong Dong’e Ejiao Food Co., Ltd. (Liaocheng, China) and stored at 2–8 °C. DPPH and FRAP assay kits (Nanjing Jiancheng Biotechnology Co., Ltd., Nanjing, China) were used to evaluate antioxidant activity. BUN and ATP assay kits (Nanjing Jiancheng Bioengineering Institute, Nanjing, China) were used to assess fatigue-related energy metabolism. MDA (Sangon Biotech Co., Ltd., Shanghai, China) and GSH-Px assay kits (Nanjing Jiancheng Bioengineering Institute, Nanjing, China) were used to determine oxidative stress status, while TNF-α, IL-1β, and IL-6 assay kits (Sangon Biotech Co., Ltd., Shanghai, China) were used to evaluate inflammatory responses.

### 2.2. Donkey Milk Polysaccharides Extraction Process

Donkey milk polysaccharides were isolated using a water extraction and ethanol precipitation method according to Li et al. [33]. Briefly, donkey milk (10 mL) was mixed thoroughly with distilled water (10 mL) and extracted in a 100 °C water bath for 2 h. After the mixture had cooled to room temperature, it was centrifuged at 10,000× *g* for 10 min. The collected supernatant (4 mL, representing the total extract from the initial 10 mL donkey milk sample) was slowly mixed with anhydrous ethanol (16 mL) and stored at 4 °C overnight for precipitation. The mixture was then centrifuged at 10,000× *g* for 10 min using a 5804 R centrifuge (Eppendorf, Hamburg, Germany), and the precipitate was collected and freeze-dried to obtain donkey milk polysaccharides.

### 2.3. Characterization of Donkey Milk Polysaccharides

#### 2.3.1. Determination of Molecular Weight

The sample was dissolved in 0.1 M NaNO_3_ aqueous solution containing 0.02% NaN_3_ (*w*/*w*) to a final concentration of 1 mg/mL, filtered through a 0.45 μm membrane filter, and analyzed using a gel permeation chromatography system (HLC-8320GPC, Tosoh Corporation, Tokyo, Japan) equipped with Ohpak SB-805 HQ (300 × 8 mm) and Ohpak SB-803 HQ (300 × 8 mm) columns connected in series. Mobile phase A (0.02% NaN_3_ in 0.1 M NaNO_3_) was used at a flow rate of 0.6 mL/min. Isocratic elution was performed for 75 min.

#### 2.3.2. Monosaccharide Composition

The monosaccharide profile of the polysaccharide fraction was determined using high-performance anion-exchange chromatography coupled with pulsed amperometric detection (HPAEC-PAD). The analysis was performed on a Dionex ICS-5000+ system (Thermo Fisher Scientific, Waltham, MA, USA) equipped with a CarboPac PA20 column (150 × 3.0 mm, 10 μm). Samples were injected at a volume of 5 μL, and chromatographic separation was conducted at a flow rate of 0.5 mL/min. Chromatographic analysis was performed with an injection volume of 5 μL and a flow rate of 0.5 mL/min. The mobile phases consisted of ultrapure water as solvent A, 0.1 M NaOH as solvent B, and 0.1 M NaOH containing 0.2 M sodium acetate (NaAc) as solvent C. The column temperature was maintained at 30 °C. The gradient elution was programmed as follows: A/B/C (*v*/*v*) ratios were 95:5:0 (0 min), 85:5:10 (26 and 42 min), 60:0:40 (42.1 min), 60:40:0 (52 min), and 95:5:0 (52.1 and 60 min).

### 2.4. In Vitro Analysis of Antioxidant Activities

DPPH radical scavenging activity and ferric reducing antioxidant power (FRAP) were determined using commercial assay kits (Nanjing Jiancheng Bioengineering Institute, Nanjing, China). Donkey milk and donkey milk polysaccharides were dissolved in distilled water (*w*/*v*) to prepare stock solutions, which were serially diluted to the indicated concentrations (donkey milk: 1, 5, 10, 20, and 50 mg/mL; donkey milk polysaccharides: 0.1, 0.5, 1, 5, and 10 mg/mL). Each concentration was tested in triplicate (*n* = 3) for both DPPH and FRAP assays.

### 2.5. Animals

**Figure 1:**
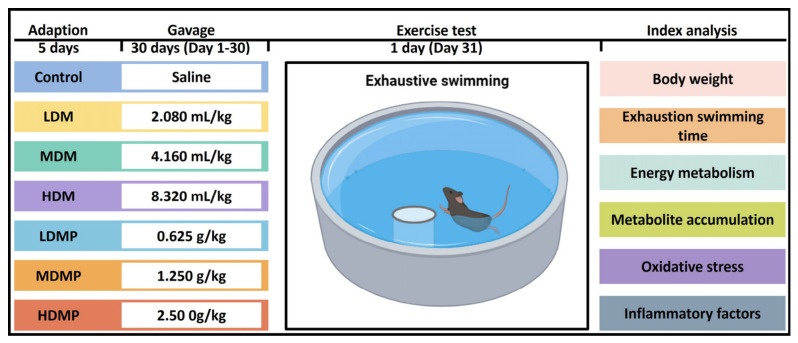
Schematic diagram of the mouse modeling experiment.

210 SPF-grade male BALB/c mice (18–22 g) were obtained from Spifo Biotechnology Co., Ltd. (Suzhou, China). The animals were housed in the Experimental Animal Center of Jiangnan University under controlled environmental conditions (temperature 20–26 °C, relative humidity 40–70%, 12 h light/dark cycle). All animal experimental procedures were approved by the Animal Ethics Committee of Jiangnan University (JN. No20250228b2000410[033]), and the facility was registered under license number SYXK (Su) 2021-0056. After a 5-day acclimatization period, the mice were randomly divided into seven groups (*n* = 30 per group): Control, low-dose donkey milk (LDM), medium-dose donkey milk (MDM), high-dose donkey milk (HDM), low-dose donkey milk polysaccharide (LDMP), medium-dose donkey milk polysaccharide (MDMP), and high-dose donkey milk polysaccharide (HDMP) (Figure 1). Donkey milk was administered orally in liquid form at doses of 2.08, 4.16, and 8.32 mL/kg body weight for LDM, MDM, and HDM groups, respectively. Donkey milk polysaccharides were dissolved in distilled water and administered orally at doses of 0.625, 1.25, and 2.5 g/kg body weight for LDMP, MDMP, and HDMP groups, respectively. Doses were determined based on human-recommended intake, converted to mouse-equivalent doses, and validated by previously reported effective doses in rodent models [22,23,32]. The gavage volume was standardized at 10 mL/kg body weight. All treatments were administered once daily for 30 consecutive days. Throughout the experiment, animals had free access to a standard chow diet and water.

ARRIVE compliance: To avoid carryover effects from repeated swimming, mice in each group were randomly allocated into three independent subgroups (*n* = 10 per subgroup) for separate experiments: weighted swimming to exhaustion, 90 min unweighted swimming followed by serum and tissue collection, and 10 min unweighted swimming for blood lactate AUC determination. All three experiments were conducted on separate days. Body weight was monitored throughout the intervention period in the subgroup assigned to the weighted swimming to exhaustion. Sample size was determined by G*Power 3.1 based on preliminary swimming time data (effect size d = 0.8, α = 0.05, power = 0.80), yielding *n* = 10 per subgroup. Investigators performing swimming tests and biochemical analyses were blinded to group allocation. Animals showing severe distress, inability to swim, or technical failure during sample collection were excluded; no animals were excluded in this study. For the weighted swimming test, the humane endpoint was defined as inability to remain above water for 10 s despite gentle stimulation; animals reaching this endpoint were immediately removed from the tank, dried, and warmed under a heat lamp.

### 2.6. Weighted Swimming Test in Mice

Thirty min after the final administration of donkey milk and donkey milk polysaccharides, the water was agitated to force the mice to swim in a swimming tank with a water depth of 35 cm and a temperature of 25 ± 1.0 °C, with a 5% body weight lead weight attached to the base of their tails. Weighted swimming time was recorded from the start of swimming until the mouse sank and remained submerged for 10 s without surfacing, marking the endpoint when limb movement ceased.

### 2.7. Determination of Biochemical Indexes Related to Anti-Fatigue Activity

#### 2.7.1. Blood Lactate Measurement Test

Blood lactate (LA) levels were determined using a BIOSEN C-Line glucose/lactate analyzer (EKF Diagnostics GmbH, Barleben, Germany) according to the manufacturer’s instructions. 30 min after the final administration of the test substance, 20 μL of blood was collected from the internal canthal venous plexus. Mice were then placed in 30 °C water for 10 min of swimming. Blood samples (20 μL) were collected from the internal angular venous plexus at 0 min and 20 min post-exercise. LA levels were measured according to the kit instructions, and the area under the LA curve was calculated.

Area under the LA curve = 1/2 × (pre-swim LA value + LA value at 0 min post-swim) × 10 + 1/2 × (LA value at 0 min post-swim + LA value at 20 min rest post-swim) × 20 = 5 × (pre-swim LA value + 3 × LA value at 0 min post-swim + 2 × LA value at 20 min rest post-swim) [34].

#### 2.7.2. Determination of Serum Biochemical Indicators

Thirty minutes after the final administration, mice in each group underwent 90 min of unweighted swimming in water maintained at 30 °C. The swimming duration and recovery period were determined according to previously reported anti-fatigue evaluation protocols. Following swimming, mice were allowed to rest for 60 min without food or electrolyte supplementation. Water was freely available during the recovery period. After anesthesia with isoflurane (3% for induction and 1.5% for maintenance), approximately 0.5 mL of blood was collected from the orbital venous plexus. Blood samples were stored at 4 °C for 3 h and centrifuged at 3000 r/min for 15 min to obtain serum. Following blood collection, mice were euthanized by cervical dislocation under isoflurane anesthesia. The liver and gastrocnemius muscle were rapidly excised, rinsed three times with ice-cold physiological saline, and stored at −80 °C for subsequent analyses. Serum lipase (LIP), lactate dehydrogenase (LDH), and superoxide dismutase (SOD) activities were measured using a Zhongyuan EC400 fully automated biochemical analyzer (Zhongyuan Biotechnology Co., Ltd., Jinan, China). Blood urea nitrogen (BUN) levels were determined using a commercial assay kit (Nanjing Jiancheng Bioengineering Institute, Nanjing, China). Serum IL-6, TNF-α, and IL-1β levels were quantified using ELISA kits (Sangon Biotech Co., Ltd., Shanghai, China). The absorbance values were measured using a Thermo 1510 microplate reader (Thermo Fisher Scientific, Waltham, MA, USA).

#### 2.7.3. Measurement of Liver-Related Indices

Hepatic glycogen (HG) content was quantified using the anthrone method and analyzed using a BIOSEN C-Line lactate/glucose analyzer (EKF Diagnostics, Barleben, Germany). Frozen liver samples were thawed on ice and homogenized in ice-cold physiological saline (1:9, *w*/*v*) using a high-speed homogenizer (Wiggens, Beijing, China) at 5000 r/min for 60 s. The homogenates were centrifuged at 5000 r/min for 15 min at 4 °C, and the supernatants were collected for subsequent assays. Protein concentration was determined using a BCA protein assay kit (Shanghai Weiwei Biological Technology Co., Ltd., Shanghai, China). Liver glutathione peroxidase (GSH-Px) activity was determined using a commercial assay kit (Nanjing Jiancheng Bioengineering Institute, Nanjing, China). Liver malondialdehyde (MDA) content was measured using a commercial assay kit (Sangon Biotech Co., Ltd., Shanghai, China). Absorbance values were recorded using a Thermo 1510 microplate reader (Thermo Fisher Scientific, Waltham, MA, USA).

#### 2.7.4. Measurement of Muscle-Related Indices

Muscle ATP levels were determined using a commercial ATP assay kit (Sangon Biotech Co., Ltd., Shanghai, China). Gastrocnemius muscle tissues were thawed on ice and homogenized with ice-cold physiological saline (1:9, *w*/*v*) using a high-speed homogenizer (5000 r/min for 60 s). After centrifugation at 5000 r/min for 15 min at 4 °C, the supernatants were collected for biochemical analyses. Absorbance was determined using a Thermo 1510 microplate reader (Thermo Fisher Scientific, Waltham, MA, USA). Absorbance values were measured using a Thermo 1510 microplate reader (Thermo Fisher Scientific, Waltham, MA, USA).

### 2.8. Data Analysis

Results were expressed as means ± standard deviation. Each biological sample was assayed in technical duplicate, and the mean value was used for statistical analysis. Statistical analyses were conducted using GraphPad Prism version 9.0.1 (GraphPad Software, San Diego, CA, USA). One-way ANOVA followed by Tukey’s multiple comparisons test was used for statistical evaluation. Data were considered statistically significant at *p* < 0.05. Figures were prepared using GraphPad Prism version 9.0.1 and Origin 2018.

## 3. Results

### 3.1. Molecular Weight and Monosaccharide Composition of Donkey Milk Polysaccharides

**Figure 2:**
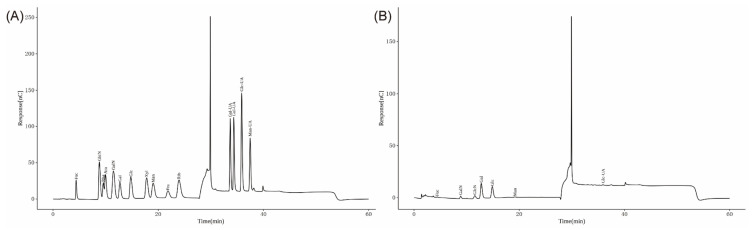
Chromatogram of monosaccharide composition. (**A**) Standard sample. (**B**) Donkey milk polysaccharides.

Donkey milk polysaccharides exhibited a broad molecular weight distribution, with an Mw/Mn value of 2.943. The number-average molecular weight (Mn), peak molecular weight (Mp), weight-average molecular weight (Mw), and Z-average molecular weight (Mz) were 14.30, 8.04, 42.08, and 229.14 kDa, respectively (Table 1). Monosaccharide composition analysis revealed that galactose was the predominant monosaccharide in donkey milk polysaccharides, accounting for 53.89 mol%, followed by glucose (28.27 mol%) and N-acetylglucosamine (9.12 mol%). Minor monosaccharides included N-acetylgalactosamine, mannose, fucose, and glucuronic acid (Figure 2).

### 3.2. In Vitro Antioxidant Activity of Donkey Milk and Donkey Milk Polysaccharides

**Figure 3:**
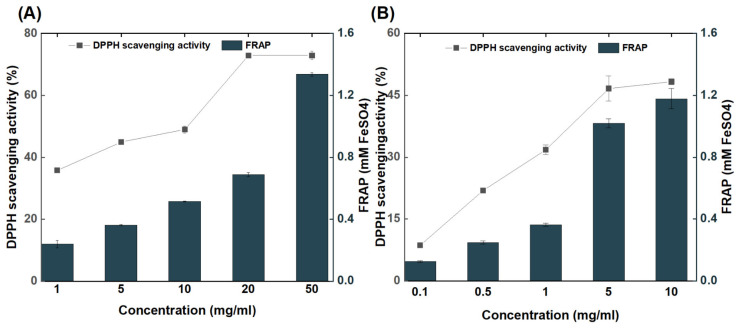
The DPPH scavenging activity and FRAP at different concentrations (*n* = 3) of (**A**) donkey milk and (**B**) donkey milk polysaccharide.

Donkey milk and donkey milk polysaccharides exhibited dose-dependent antioxidant activities in vitro, as supported by increased DPPH radical scavenging activity and FRAP values (Figure 3). Donkey milk plateaued at 20.00 mg/mL, achieving a maximum scavenging efficiency of 72.88 ± 0.24%. Donkey milk polysaccharide fraction plateaued at 5.00 mg/mL with 46.68 ± 3.08% efficiency. FRAP increased linearly with concentration for both samples, with the donkey milk polysaccharide fraction displaying significantly higher FRAP values than donkey milk at each equivalent concentration. Donkey milk and donkey milk polysaccharides exhibited concentration-dependent antioxidant activity in vitro (Figure 3).

### 3.3. Body Weight of the Mice

Body weight increased gradually in all groups over the 30-day intervention period (Table 2). After 30 days of daily gavage with donkey milk and donkey milk polysaccharides, no significant intergroup differences in body weight were detected relative to the Control (*p* > 0.05).

### 3.4. Effects of Donkey Milk and Donkey Milk Polysaccharides on the Weight-Bearing Swimming Test in Mice

**Figure 4:**
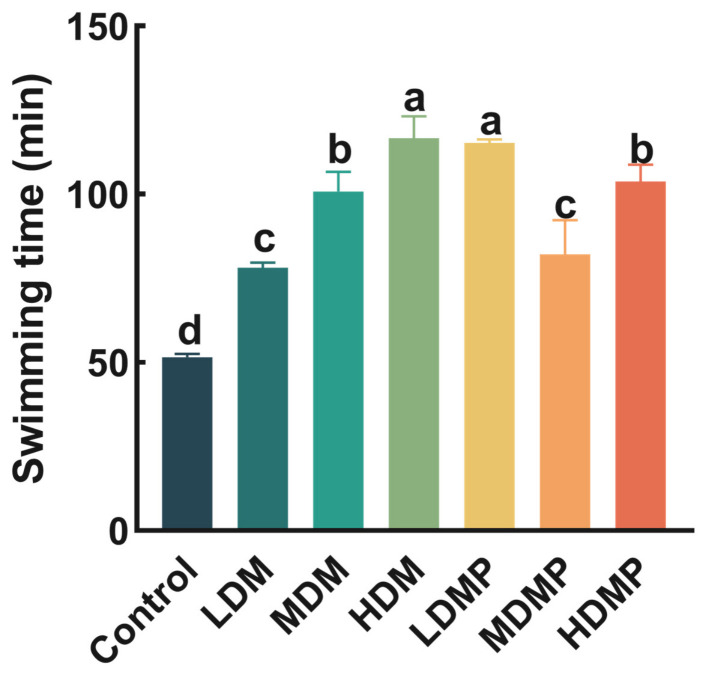
Effects of donkey milk and donkey milk polysaccharides on swimming time in mice. Note: Data are presented as mean ± SD (*n* = 10). Statistical analysis was performed using one-way ANOVA followed by Tukey post-tests. Different letters indicate significant differences between groups (*p* < 0.05).

Exhaustive swimming time was significantly prolonged in all donkey milk and donkey milk polysaccharide treatment groups compared with the Control group (51.50 ± 1.05 min, *p* < 0.05) (Figure 4). Among the treatment groups, HDM (116.67 ± 6.50 min) and LDMP (115.33 ± 1.03 min) exhibited the longest exhaustive swimming times, corresponding to 2.27-fold and 2.25-fold increases relative to the Control group, respectively (*p* < 0.05).

### 3.5. Effects of Donkey Milk and Donkey Milk Polysaccharides on Energy Metabolism in Mice

**Figure 5:**
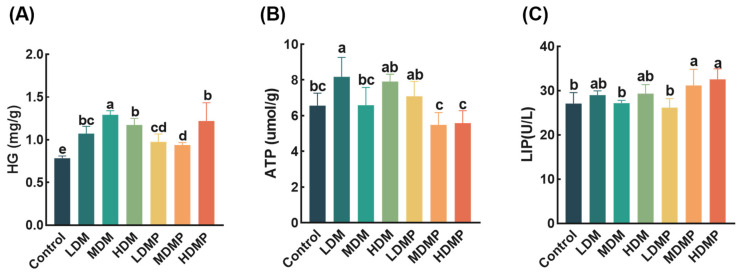
Effects of donkey milk and its polysaccharides on energy metabolism indicators in mice; (**A**) HG content; (**B**) ATP content; (**C**) LIP activity. Note: Data are presented as mean ± SD (*n* = 10). Statistical analysis was performed using one-way ANOVA followed by Tukey post-tests. Different letters indicate significant differences between groups (*p* < 0.05). Abbreviations: HG, hepatic glycogen; ATP, adenosine triphosphate; LIP, lipase.

In mice subjected to 90 min swimming, post-exercise HG reserves were significantly elevated by both donkey milk and donkey milk polysaccharides relative to the Control (Figure 5A; *p* < 0.05). MDM produced the largest increment (66.67%, *p* < 0.05). Skeletal muscle ATP (Figure 5B) was significantly higher in LDM and LDMP than in Control (*p* < 0.05), whereas HDM also exceeded Control (*p* < 0.05) and the remaining treatments were statistically equivalent (*p* > 0.05). LIP activity did not differ between donkey milk treatments and Control (*p* > 0.05; Figure 5C). In contrast, MDMP and HDMP exhibited significantly elevated lipase activity relative to Control (*p* < 0.05). These data indicate that donkey milk and donkey milk polysaccharides improved post-exercise energy status, with effects varying by dose and component.

### 3.6. Effects of Donkey Milk and Donkey Milk Polysaccharides on Metabolite Accumulation in Mice

**Figure 6:**
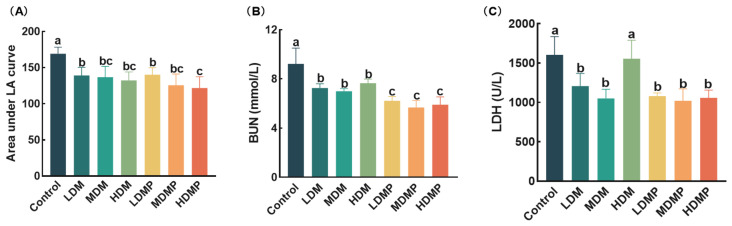
Effects of donkey milk and donkey milk polysaccharides on serum metabolites in mice after exercise; (**A**) area under the blood lactate curve; (**B**) BUN content; (**C**) LDH activity. Note: Data are presented as mean ± SD (*n* = 10). Statistical analysis was performed using one-way ANOVA followed by Tukey post-tests. Different letters indicate significant differences between groups (*p* < 0.05). Abbreviations: BUN, blood urea nitrogen; LDH, lactate dehydrogenase.

In the swimming experiment, both interventions significantly reduced the blood lactate AUC (Figure 6A), BUN content (Figure 6B), and LDH activity (Figure 6C) (*p* < 0.05) relative to the Control group. Given the superior reductions in LA AUC (17.15–28.14%), BUN (32.71–38.50%), and LDH (32.61–36.42%) relative to donkey milk (LA AUC, 17.71–21.78%; BUN, 17.08–24.18%; LDH, 3.02–34.54%), the polysaccharide fraction is likely the key contributor to attenuating serum metabolite accumulation.

### 3.7. Effects of Donkey Milk and Donkey Milk Polysaccharides on Oxidative Stress in Mice

**Figure 7:**
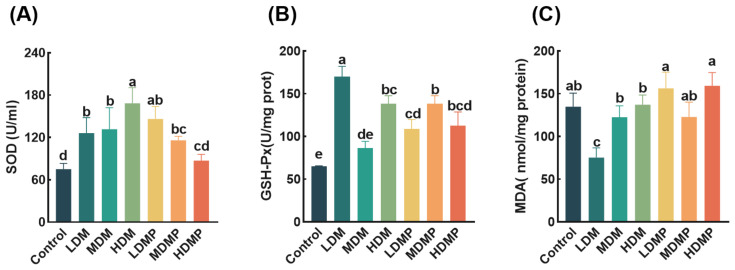
Effects of donkey milk and its polysaccharides on oxidative stress markers in mice; (**A**) SOD activity; (**B**) GSH-Px activity; (**C**) MDA content. Note: Data are presented as mean ± SD (*n* = 10). Statistical analysis was performed using one-way ANOVA followed by Tukey post-tests. Different letters indicate significant differences between groups (*p* < 0.05). Abbreviations: SOD, superoxide dismutase; GSH-Px, glutathione peroxidase; MDA, malondialdehyde.

Figure 7 summarizes oxidative stress biomarkers across experimental groups. Both donkey milk and donkey milk polysaccharides significantly elevated SOD (Figure 7A) and GSH-Px (Figure 7B) activities relative to Control (*p* < 0.05). HDM (168.50 ± 22.31 U/mL) and LDMP (146.30 ± 17.53 U/mL) achieved the highest SOD activities, whereas LDM (170.10 ± 11.96 U/mg prot) exhibited the greatest GSH-Px activity (*p* < 0.05). MDA concentration (Figure 7C) was significantly reduced only in LDM (75.48 ± 11.25 nmol/mg protein) relative to Control (134.83 ± 15.99 nmol/mg protein; *p* < 0.05). All remaining groups were statistically equivalent to Control (*p* > 0.05).

### 3.8. Effects of Donkey Milk and Donkey Milk Polysaccharides on Inflammation in Mice

**Figure 8:**
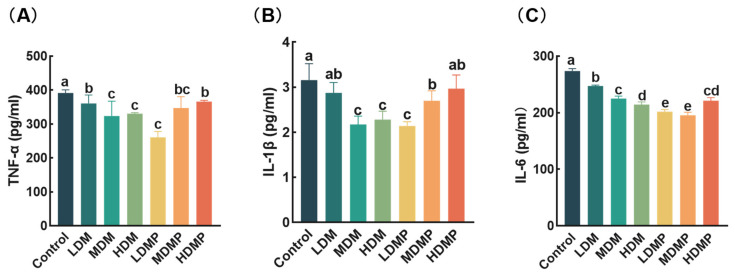
Effects of donkey milk and its polysaccharides on inflammatory factors in mice; (**A**) TNF-α content; (**B**) IL-1β content; (**C**) IL-6 content. Note: Data are presented as mean ± SD (*n* = 10). Statistical analysis was performed using one-way ANOVA followed by Tukey post-tests. Different letters indicate significant differences between groups (*p* < 0.05). Abbreviations: TNF-α, tumor necrosis factor-alpha; IL-1β, interleukin-1 beta; IL-6, interleukin-6.

In the present model, exercise-induced fatigue markedly elevated circulating TNF-α, IL-1β, and IL-6 concentrations. Relative to the Control, donkey milk and donkey milk polysaccharide interventions significantly suppressed these cytokines (Figure 8; *p* < 0.05). Among DM, HDM yielded the lowest TNF-α (Figure 8A) and IL-1β (Figure 8B) concentrations (330.70 ± 2.86 pg/mL and 2.17 ± 0.19 pg/mL, respectively; *p* < 0.05) and reduced TNF-α, IL-1β, and IL-6 (Figure 8C) by 15.50%, 27.76%, and 21.70% versus Control. Among DMP, LDMP produced the most pronounced suppression (*p* < 0.05). LDMP decreased TNF-α to 260.90 ± 17.31 pg/mL (33.33%), IL-1β to 2.14 ± 0.10 pg/mL (32.26%), and IL-6 to 202.00 ± 3.72 pg/mL (26.20%).

## 4. Discussion

**Figure 9:**
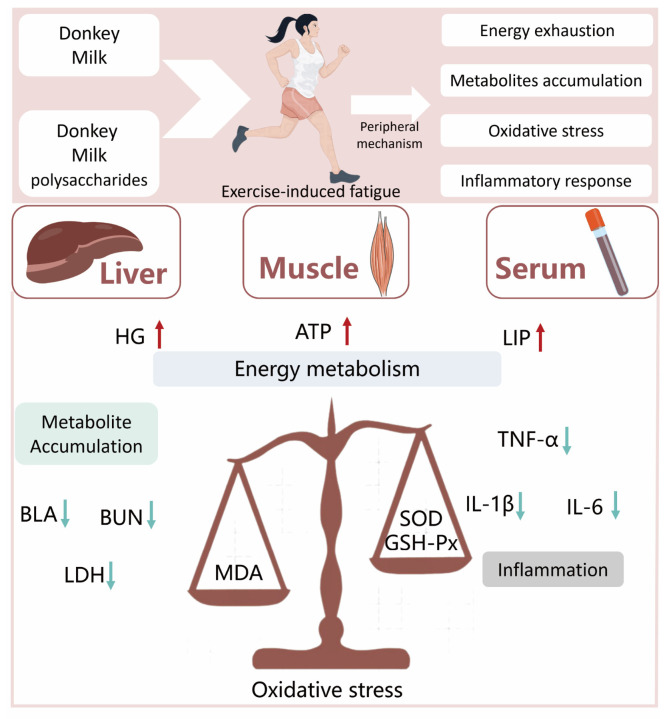
Integrated model of energy metabolism, oxidative stress, and inflammation in the anti-fatigue effects of donkey milk polysaccharides.

Exercise-induced fatigue is a multifactorial syndrome driven by energy depletion, oxidative stress, and inflammation, which together compromise endurance performance and delay recovery (Figure 9). This study systematically evaluated the differential anti-fatigue effects of donkey milk and its polysaccharides by integrating polysaccharide composition analysis, in vitro antioxidant assessment, and in vivo anti-fatigue evaluation. Notably, the experimental framework established a potential relationship across polysaccharide compositional features, in vitro bioactivity, and in vivo physiological outcomes, enabling a coherent interpretation of structure–function relationships. Polysaccharide composition analysis revealed that donkey milk polysaccharides are predominantly composed of galactose (Gal), consistent with previous reports [16,17,35]. Gal is not only an efficient energy substrate that can enter glycolytic and glycogenic pathways after conversion to glucose-1-phosphate, but also a critical monosaccharide for the biosynthesis of glycoproteins and glycolipids, thereby contributing to cellular redox homeostasis and membrane stability. The high galactose content may therefore partly explain the metabolic and antioxidant benefits observed in vivo [36], providing a structural basis for linking composition with functional outcomes. In vitro assays indicated that both donkey milk and its polysaccharides possess concentration-dependent DPPH radical scavenging and ferric reducing antioxidant power (FRAP) assays (Figure 3). Notably, donkey milk showed relatively stronger DPPH radical scavenging activity, whereas the polysaccharide fraction exhibited greater ferric reducing antioxidant power. Consistent with previous reports demonstrating the antioxidant potential of polysaccharides [37], these findings suggest that the compositional features of donkey milk polysaccharides are closely associated with their intrinsic antioxidant capacity, highlighting the relationship between compositional features and biological activity.

Decreased exercise endurance is the most overt indicator of physical fatigue [38]. The exhaustive swimming test is widely used in anti-fatigue research because of its objectivity and reproducibility [39]. Accordingly, anti-fatigue efficacy was assessed in a mouse exhaustive swimming model. Both donkey milk and donkey milk polysaccharides significantly prolonged exhaustive swimming time, with HDM and LDMP displaying the greatest efficacy (Figure 4). Body weight gain remained normal and did not differ significantly from Control across all treatment groups (Table 2). Absence of clinical abnormalities or mortality indicates that both interventions enhanced endurance at non-toxic doses, corroborating earlier reports [22,23].

Energy depletion is a primary determinant of exercise-induced fatigue. HG maintains blood glucose homeostasis during exercise, whereas ATP provides the immediate energy for muscle contraction [40]. Donkey milk and donkey milk polysaccharides significantly elevated HG stores and preserved skeletal muscle ATP in exercise-fatigued mice (Figure 5A and Figure 5B, respectively). MDM produced the greatest gain in hepatic glycogen, whereas LDM, HDM, and LDMP were most effective in sustaining muscle ATP. These findings indicate that fatigue onset is delayed through optimization of energy supply, corroborating reports that polysaccharides augment energy reserves [41,42,43]. In addition to modulating glycogen and ATP metabolism, donkey milk polysaccharides also influence lipid utilization. LIP, a key lipolytic enzyme, was significantly up-regulated by MDMP and HDMP (Figure 5C). This may reflect altered lipid metabolism, which could contribute to energy substrate diversification, though direct evidence of enhanced fatty acid oxidation was not obtained. Collectively, these metabolic adaptations support the energetic foundation for alleviating exercise-induced fatigue [44].

Donkey milk and donkey milk polysaccharides accelerate post-exercise metabolic waste clearance, restore internal homeostasis, and alleviate metabolic fatigue. Both interventions decreased the area under the LA AUC, BUN concentration, and LDH activity in exercise-fatigued mice (Figure 6). The observed reductions in LA AUC, BUN, and LDH are consistent with decreased metabolic stress in exercised mice. Lower BUN levels may suggest reduced protein catabolism under exercise stress, while reduced LDH activity may reflect decreased cellular stress [45,46]. Donkey milk polysaccharides produced a significantly greater decrement in both BUN and LDH than donkey milk, implicating them as the principal regulators of nitrogen metabolism and cellular protection [32].

Oxidative stress is a major driver of exercise-induced fatigue; excessive reactive oxygen species disrupt redox homeostasis [47,48]. Elevated free radicals increase lipid peroxidation, thereby impairing cellular metabolism and precipitating muscle weakness [3]. SOD and GSH-Px constitute the primary enzymatic defense: SOD dismutates superoxide (O_2_•^−^) to hydrogen peroxide (H_2_O_2_), which GSH-Px subsequently reduces to water and lipid hydroperoxides to alcohols [49,50]. MDA, the terminal product of lipid peroxidation, quantifies oxidative damage [51]. Donkey milk and donkey milk polysaccharides significantly elevated serum SOD and GSH-Px activities (Figure 7A,B) in exercise-fatigued mice, thereby enhancing endogenous antioxidant capacity. HDM and LDMP, which exhibited the highest SOD activities, also displayed the longest exhaustive swimming times, implying a positive association between SOD-mediated antioxidant capacity and endurance performance [52]. Although antioxidant enzyme activities were universally elevated, MDA declined significantly only in the LDM group (Figure 7C). This suggests that donkey milk and donkey milk polysaccharides modulate oxidative stress via additional pathways beyond SOD and GSH-Px to alleviate physical fatigue.

Exercise-evoked inflammation accelerates both the onset and progression of fatigue. Elevated pro-inflammatory cytokines, specifically TNF-α, IL-1β, and IL-6, correlate closely with muscle damage and delayed recovery [51]. Donkey milk and donkey milk polysaccharides significantly decreased serum concentrations of these cytokines in exercise-fatigued mice, with HDM and LDMP displaying the most pronounced inhibition (Figure 8). These findings demonstrate that donkey milk and donkey milk polysaccharides are associated with reduced pro-inflammatory cytokine levels, corroborating their supported immunomodulatory and anti-inflammatory properties [15,24]. Collectively, these results support an integrated mechanistic framework in which donkey milk polysaccharides exert antioxidant effects that are associated with improved redox homeostasis, energy metabolism, and inflammatory regulation, ultimately contributing to enhanced exercise endurance. Further studies are required to elucidate the underlying molecular mechanisms.

However, several limitations of this study should be acknowledged. First, the absence of a non-exercised (sedentary) control group precludes direct attribution of biochemical changes to exercise alone; therefore, our findings reflect differences between treated and untreated exercised mice rather than exercise-induced changes per se. Second, the polysaccharide fraction was characterized by molecular weight and monosaccharide composition only; extraction yield, purity, and residual protein or lactose content were not quantified, which may limit the interpretability of structure–activity considerations. Third, the mouse swimming model, while widely used, has limited translational validity to human fatigue management. Finally, the observed biomarker changes suggest associations rather than confirmed mechanistic pathways. Future studies using purified polysaccharide fractions together with targeted molecular pathway analyses will be necessary to further elucidate the biological mechanisms underlying the observed anti-fatigue effects.

## 5. Conclusions

This study demonstrated that donkey milk polysaccharides, characterized by a broad molecular weight distribution and high galactose content, exhibited in vitro antioxidant activity. The antioxidant effects identified in vitro were consistent with the improved physiological outcomes observed in vivo, indicating a functional link between bioactivity and anti-fatigue effects. Both donkey milk and its polysaccharides prolonged exhaustive swimming time and improved fatigue-related biochemical markers in exercised mice, including energy metabolites, oxidative stress indicators, and inflammatory cytokines. These findings suggest that donkey milk and its polysaccharides may serve as potential functional ingredients for fatigue management, though further mechanistic studies are warranted.

## Figures and Tables

**Table 1:** Molecular weight of donkey milk polysaccharides.

	Mn (kDa)	Mp (kDa)	Mw (kDa)	Mz (kDa)	Polydispersity (Mw/Mn)
Donkey milk polysaccharides	14.299	8.042	42.083	229.138	2.943

**Table 2:** Effect of DM and DMP on the body weight of mice.

Groups	Initial Weight (g)	Final Weight (g)
Control	20.99 ± 0.54	24.88 ± 1.36
LDM	20.63 ± 0.81	24.57 ± 2.36
MDM	20.71 ± 0.64	24.74 ± 1.39
HDM	20.74 ± 0.90	25.40 ± 1.32
LDMP	21.03 ± 0.82	23.59 ± 0.71
MDMP	20.27 ± 0.89	24.84 ± 0.96
HDMP	20.56 ± 0.60	24.19 ± 1.35

Note: Data are presented as mean ± SD (*n* = 10). No significant differences were detected between groups at any time point (*p* > 0.05). Abbreviations: DM, donkey milk; DMP, donkey milk polysaccharide.

## Data Availability

The original contributions presented in the study are included in the article; further inquiries can be directed to the corresponding authors.
